# Defining the information needs of lung cancer screening participants: a qualitative study

**DOI:** 10.1136/bmjresp-2019-000448

**Published:** 2019-11-24

**Authors:** Mamta Ruparel, Samantha Quaife, David Baldwin, Jo Waller, Samuel Janes

**Affiliations:** 1 Lungs for Living Research Centre, UCL Respiratory, University College London, London, UK; 2 Research Department of Behavioural Science and Health, University College London, London, UK; 3 Respiratory Medicine Unit, David Evans Research Centre, Nottingham University Hospitals NHS Trust, Nottingham, UK; 4 Cancer Prevention Group, King's College London, London, UK

**Keywords:** lung cancer, imaging/CT MRI etc

## Abstract

**Introduction:**

Lung cancer screening (LCS) by low-dose CT has been shown to improve mortality, but individuals must consider the potential benefits and harms before making an informed decision about taking part. Shared decision-making is required for LCS in USA, though screening-eligible individuals’ specific views of these harms, and their preferences for accessing this information, are not well described.

**Methods:**

In this qualitative study, we aimed to explore knowledge and perceptions around lung cancer and LCS with a focus on harms. We carried out seven focus groups with screening-eligible individuals, which were divided into current versus former smokers and lower versus higher educational backgrounds; and 16 interviews with health professionals including general practitioners, respiratory physicians, lung cancer nurse specialists and public health consultants. Interviews and focus groups were audio-recorded and transcribed. Data were coded inductively and analysed using the framework method.

**Results:**

Fatalistic views about lung cancer as an incurable disease dominated, particularly among current smokers, and participants were often unaware of curative treatment options. Despite this, beliefs that screening is sensible and worthwhile were expressed. Generally participants felt they had the ‘right’ to an informed decision, though some cautioned against information overload. The potential harms of LCS were poorly understood, particularly overdiagnosis and radiation exposure, but participants were unlikely to be deterred by them. Strong concerns about false-negative results were expressed, while false-positive results and indeterminate nodules were also reported as concerning.

**Conclusions:**

These findings demonstrate the need for LCS information materials to highlight information on the benefits of early detection and options for curative treatment, while accurately presenting the possible harms. Information needs are likely to vary between individuals and we recommend simple information materials to be made available to all individuals considering participating in LCS, with signposting to more detailed information for those who require it.

**Key messages**

- Education around the potential curability of early stage lung cancer is vital.
- Preferences for information provision varied: individuals wished to be fully informed but some also cautioned against information overload. Strong concerns about false-negative results were expressed, while false positives and indeterminate nodules were also reported as concerning.
- Our data represent the preferences of at-risk individuals for information needed to support informed choice; a perspective which should inform approaches to screening implementation. We propose that key information should be presented simply without overburdening individuals, but with further detailed information readily available to suit individuals' preferences.

## Introduction

Lung cancer screening (LCS) by low-dose CT (LDCT) reduces mortality. The US National Lung Screening Trial showed annual LDCT of high-risk adults for three years reduced lung cancer-specific mortality by 20% compared with chest radiograph^1^ and this positive effect has been further confirmed in two further randomised studies.^2 3^

LDCT screening also poses potential harms through overdiagnosis, false-positive findings, clinically insignificant incidental findings, low-dose radiation exposure and psychological burden.^4^ Individuals who take part in screening may have to live with the negative consequences of the screening test. Even if the ratio of harm to benefit is low, individuals must understand and weigh up the risks and benefits in order to make an informed decision to participate.^5^ A participant-centred approach focused on enhancing this process may reduce the psychological burden of harms.^6^ The US Centre for Medicare and Medicaid services mandates a shared decision-making process for individuals undergoing LCS:^7^ while ‘shared’ and ‘informed’ decision-making are distinct concepts, both are in line with moves towards informed choice and greater participant involvement, which are employed across the cancer screening context.^5^ Meanwhile in the UK in 2005, the National Screening Committee concluded there was insufficient evidence to support LCS, a review of which was due in 2015 and is yet awaited.^8^ Nevertheless a number of small-scale pilots are underway,^9 10^ as well as a larger-scale National Health Service England-led initiative to be implemented across 14 sites around England.^11^ Provision of adequate information to individuals considering taking part needs to be a high priority in the roll-out of LCS in the UK.

There is good evidence from psychological science about how best to present risk information in the medical context^12^ but less consensus about what constitutes an informed choice in cancer screening.^13^ Overdiagnosis is an aspect of screening information that has proved particularly challenging to communicate,^14^ although there is strong evidence that women participating in mammography screening want to be informed about this risk.^15 16^ Optimising communication about harms and benefits is particularly pertinent in lung cancer, where those from lower socioeconomic backgrounds, who often have lower levels of literacy and numeracy,^17^ make up a large proportion of the highest-risk population.^18^ Alongside best evidence on risk communication, the preferences of the target population are an important consideration in developing information materials for a new screening test. At present, very little is known about the information preferences of potential lung screening invitees. Therefore, we carried out an in-depth qualitative study using focus groups and interviews with a lung cancer ‘at-risk’ population and healthcare professionals (HCPs) to address the following research questions: what do LCS-naïve individuals from an ‘at-risk’ population and HCPs involved in lung cancer and public health (PH) services believe LCS participants (1) Know and perceive about lung cancer treatment. (2) Know, perceive and want to know about LCS (including harms and benefits).

## Methods

### Participants

#### Focus groups

Participants were purposively recruited from three general practice (GP) surgeries in an ethnically diverse area of London with no LCS. An electronic record search identified patients aged 60–75 years who had been recorded as current smokers within the past 15 years to identify those likely to be eligible for LCS, mimicking our LDCT demonstration pilot the Lung Screen Uptake Trial.^9^ An invitation letter was sent on behalf of the participant’s primary care physician. The invitation pack included a participant information sheet for the study, and an option to opt out of being contacted by the research team. A member of the research team (MR) phoned those who had not opted out and collected data on smoking history (status, years smoked and average cigarette consumption per day) and demographic details (age, sex, education level, ethnicity, religion). This enabled individuals who met the US Preventative Services Task Force (USPSTF) criteria for LCS, and who agreed to participate, to be allocated to a focus group based on smoking status and educational level. Sample size was determined on the attainment of data saturation (lack of emergence of any new themes), with scope for a further phase of recruitment if required.

#### Interviews

We recruited a variety of HCPs for two principal reasons. First, we valued the professional insights and opinions of HCPs who have experience of working with patients at risk of lung cancer and with lung cancer at various points in their patient journey. This provided an additional and important dimension to our understanding of the information needs of potential LCS participants. Second, we felt that as with any qualitative study, a degree of selection bias was likely. By including data from HCPs (who could draw on their clinical experience, including with individuals that may be less likely to engage with LCS) we hoped to overcome some of this bias and make the combined HCP and focus group participant data more reflective of the range of views in the target population. Recruited HCPs were from four disciplines (GPs, lung cancer nurse specialists (CNS), respiratory physicians (RP) and public health (PH) consultants), using snowballing. The target sample size was attained when three or more participants from each discipline were recruited and when data saturation (ie, the lack of emergence of any new themes) was achieved.

### Patient and public involvement

Prior to embarking on the study we carried out some patient and public involvement work by piloting screening materials containing the relevant information required to make an informed decision on LCS, on members of the public from the desired age group. This directly led to the development of the design and protocol for the current study.

### Data collection

#### Focus groups

The focus groups were carried out by MR (a female research fellow with a background in respiratory medicine), in May 2016 in a local library and lasted 90 min each. Written consent was obtained at the start of the groups. The sessions were run by two facilitators (one to facilitate the discussion and one to observe and make notes), and were audio-recorded.

Open discussion between participants was encouraged and facilitated, with more narrow questions where needed to address the research questions as described in the discussion guide (online supplementary appendix 1). The facilitator provided some verbal (box 1) and written information (using existing US and UK information materials) on LCS. Participants could ask questions and were encouraged to give feedback and opinions on lung cancer and screening, LCS harms and benefits, information materials and smoking cessation in the context of LCS. The interviewer did not reveal her background or any personal views or goals about the research to the participants.

10.1136/bmjresp-2019-000448.supp1 Supplementary data

Box 1 **Description focus group participants were given about lung cancer screening during the discussion**

‘Research in the US has shown that if we carry out a CT scan (a detailed sort of X-ray) once a year on people who have a higher risk of lung cancer due to the amount they have smoked in the past, we may save 20% of lives by detecting the cancer early and giving a higher chance of cure. There are more trials underway, and depending on the results of those, we may start doing lung cancer screening in the UK in a few years. As with the other screening programmes we have discussed, there are pros and cons to screening for lung cancer. Here are some leaflets on lung cancer screening. I will give you some time to read through them and then, if it’s ok, I’ll ask you for your thoughts on them.‘

#### Interviews

Telephone interviews were carried out by MR to enable professionals (in rural and urban practice) to participate and were conducted from April to June 2016. Participants were sent information about the study and a consent form by email. Interviews lasted between 25 min and 50 min and were audio-recorded.

The interview schedule (online supplementary appendix 2) followed a similar structure to the focus group discussion guide. Participants were asked about their views on patients’ perceptions of lung cancer, the harms and benefits of screening, their experiences of communicating complex facts or statistics and smoking cessation.

### Analysis

Focus groups and interviews were transcribed verbatim (ie. exactly as spoken) by a professional transcription service. MR coded the data inductively and SQ second-coded >10% of focus group transcripts using the same coding framework. The codes were collated, organised into themes and analysed using the matrix-based framework method,^19 20^ with themes in the columns and participants in the rows. This allowed examination of focus group data by educational background and smoking status. The framework was discussed with coauthors SQ and JW and the transcripts were re-reviewed following the group discussion. Analysis continued into the write-up phase. Coding was carried out using NVivo V.11.

## Results

**Figure 1:**
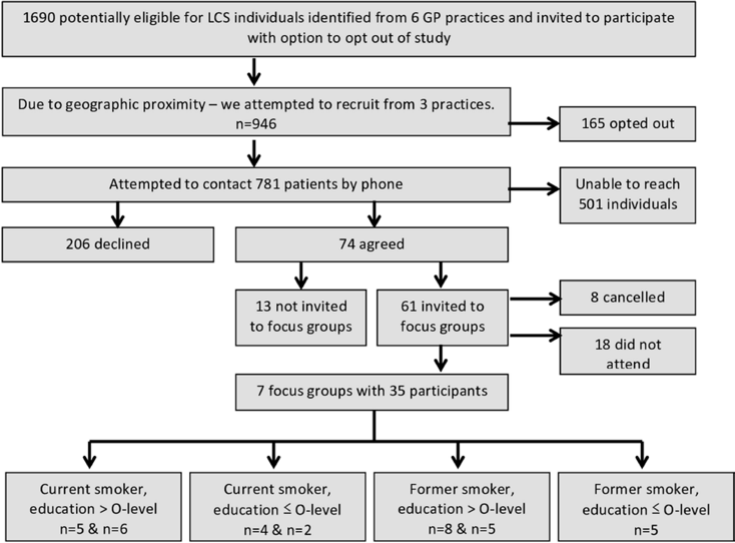
Process of recruitment for the focus groups. GP, general practice; LCS, lung cancer screening.

Of the 946 individuals identified and invited from three GP practices, 280 individuals were successfully contacted by phone and 74 (26.4%) agreed to take part in the study. Of the 61 (22.9%) individuals allocated and invited to a focus group, 35 (12.5%) individuals participated in seven focus groups (figure 1). The demographic and smoking-related characteristics of participants are described in table 1. Of the 18 HCP participants, 7 were GPs (some of whom had a special interest in cancer), 4 were lung CNS, 4 were respiratory physicians and 3 were public health consultants.

**Table 1:** Focus group participant characteristics (% totals may not sum up due to rounding)

n (%)
Gender	
Male	19 (54%)
Female	16 (46%)
Age, median (IQR)	
Age, years	66 (62, 70)
Ethnicity, n (%)	
White British	26 (74%)
White European	2 (6%)
White Irish	1 (3%)
Black Jamaican	1 (3%)
British Indian	1 (3%)
Kurdish	1 (3%)
Iranian	1 (3%)
Somalian	1 (3%)
Prefers not to say	1 (3%)
Religion, n (%)	
Christian	13 (37%)
None	12 (34%)
Jewish	6 (17%)
Muslim	2 (6%)
Hindu	1 (3%)
Other	1 (3%)
Education: education ≤O-level groups, n (%)	
Finished school at or before the age of 15 years	7 (20%)
Completed CSEs, O-levels or equivalent	4 (11%)
Completed A-levels of equivalent	1 (3%)
Completed further education but not a degree	11 (31%)
Completed a Bachelor's degree	9 (26%)
Completed a further degree (eg, Masters/ PhD)	3 (9%)
Smoking status, n (%)	
Current smoker	17 (49%)
Former smoker	18 (51%)
Smoking: current smoker groups, median (IQR)	
Years smoked	49 (41,54)
Average smoked per day, cigarettes/day	20 (10, 25)
Smoking pack-years	44 (27, 51)
Smoking: former smoker groups, median (IQR)	
Years smoked	37 (24, 42)
Average smoked per day, cigarettes/day	20 (12, 30)
Smoking pack-years	38 (18, 50)

CSE, Certificate of Secondary Education; IQR, Inter-Quartile Range; PhD, Doctor of Philosophy.

### Themes

**Figure 2:**
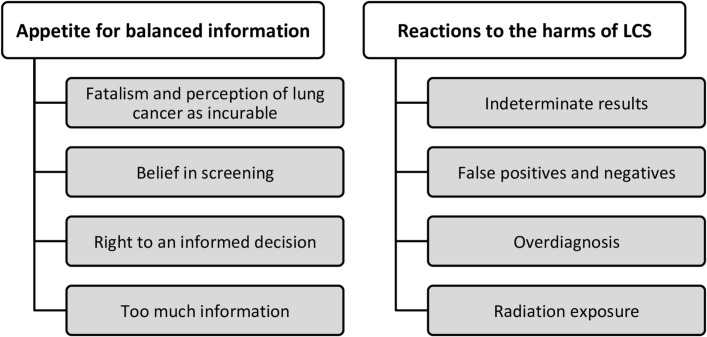
Thematic structure. LCS, lung cancer screening.

Two general themes were interpreted to address the research questions for both the focus group and interview data, each with a number of subthemes (figure 2). Theme 1 contains four subthemes, which provide insight into what general information may be included in LCS information materials, what points should be highlighted and how this may be best presented. Theme 2 describes the opinions of the focus group participants and HCPs on the various possible harms associated with LCS (four subthemes). Below are descriptions of each subtheme with illustrative extracts: focus group participants (FG) are denoted by gender (M/F), smoking status (CS, current smoker /FS, former smoker) and education descriptor (ED+, education >O-level/ED−, education ≤O-level). The interview participants (INT) are denoted by their professional roles: GP (general practitioner)/RP (respiratory physician)/CNS (lung cancer nurse specialist)/PH (public health consultant) from the data (also tables 2 and 3).

**Table 2:** Quotes illustrating the 'Appetite for balanced information' theme

Theme 1: ‘appetite for balanced information’	
**Fatalism and perception of lung cancer as incurable**	
*‘they were 99.9% certain they’d got rid of all the cancer … but 3 weeks later it was back‘*	F5, FG67_FS_ED−
*‘I thought they didn’t do surgery for lung cancer’*	F2, FG63_CS_ED−
*‘My father had it (lung cancer*), *but really in the ‘70 s so it was… quite new, it wasn’t a new cancer but it was… to survive it, and he survived it’*	F4, FG67_FS_ED−
**Belief in screening**	
*‘I think the message … for things like breast cancer and bowel cancer … is that people can actually be cured from it … I think most people see lung cancer as something which isn’t going to be cured’*	INT38_GP
*‘I mean people who throw it (bowel screening kit) away I don’t understand, quite frankly, for the effort it takes. Any prevention is better than cure’*	M6, FG63_CS_ED+
*‘any screening that’s ever offered, I think anybody that doesn’t take it must be completely bonkers. I mean why wouldn’t you? If it’s offered. You need to know. And as early as possible’*	F7, FG64_FS_ED+
*‘generally people want the test and it’s you saying to them, well actually you need to understand that we’ve got to have a good reason to do it because we might pick up things that are not helpful’*	INT38_GP
**Right to an informed decision**	
*‘you want to make a decision that’s an informed one … Not one where you say afterwards, oh, I didn’t know that … I just want to be told the full facts’*	F3, FG68_CS_ED−
*‘I think now this is a concept in general practice of giving people the facts in a way that they can understand so that they can make that shared decision’*	INT61_GP
**Too much information**	
*‘There’s also an argument that says, do you really want to … access the information, because sometimes the information can be more scary (than the disease*)’	M6, FG70_CS_ED+
*‘Oh, they manipulate everything these days to suit themselves … Because, because everybody from the Government, all the way down, massage things to suit whatever they’re doing at that time’*	F4, FG63_CS_ED+

CS, current smoker; ED, education; F, female; FG, focus group participants; FS, former smoker; GP, general practice; INT, interview participant; M, male.

**Table 3:** Quotes illustrating the ‘reactions to the harms of LCS’ theme

Theme 2: ‘reactions to the harms of LCS’	
**Indeterminate pulmonary nodules**	
*‘They would say things like I'm glad it, I'm glad this nodule hasn’t changed and I have been a bit worried about it but at least it was found and at least somebody’s looking after it’*	INT45_RP
*‘(indeterminate results) can cause a lot of anxiety and that’s where the clinicians role is, to reassure them and that can take some time’*	INT72_RP
**False negatives and false positives**	
*‘I think the biggest problem is when they do a test and they say, oh, yes, you’re fine, and in actual fact, it didn’t pick up the problem … a false positive is probably OK because they … they’ll have a, probably a backup test … or they’ll do the test again’*	M3, FG65_FS_ED+
*‘I’ve actually known that happen to someone where they got a letter through the post saying everything was OK and 6 weeks later they were dead’*	M4, FG68_CS_ED−
**Overdiagnosis**	
*‘I still think I’d go the whole hog just so that I could still be alive and say, OK, so it wasn’t that necessary but… you should because I’m still here’*	F1, FG68_CS_ED−
*‘Well, I think he (my father) took to it (watching and waiting) OK. I think the rest of the family were going, so he’s saying he’s got cancer and he doesn’t want to do anything about it. … has my father not been assertive enough? Has the doctor just said, go away, we’re not bothered?’*	M2, FG70_CS_ED+
**Radiation exposure**	
*‘Well it doesn’t worry me. You know, it’s just one of those things that have got to be done’*	M6, FG64_FS_ED+
*‘I think radiation is particularly difficult because people are, either just ignore it because they don’t understand it or they become very, very anxious about it because they don’t understand it’*	INT46_PH

CS, current smoker; ED, education; F, female; FG, focus group participant; FS, former smoker; INT, interview participant; LCS, lung cancer nurse specialist; M, male; PH, public health consultant; RP, respiratory physician.

#### Theme 1: appetite for balanced information (table 2)

##### Fatalism and perception of lung cancer as incurable

Many participants described lung cancer with terms such as *‘death sentence’* (INT54_CNS) or *‘death knell’* (M6, FG64_FS_ED+). Focus group participants, most commonly smokers, expressed views that it had poor prognosis, worse than other cancers and this was echoed by the HCPs. One smoker stated that they were ‘wary’ (F2, FG63_CS_ED+) of LCS given the poor prognosis of lung cancer.

Many participants were unaware of curative treatment options. Some had encountered surgery, but still associated this with negative outcomes, such as cancer recurrence. A few recounted stories of positive surgery outcomes, but cited that this was unusual.

The HCPs also described a lack of public awareness of curative treatments and talked about smokers being *‘extremely worried about lung cancer’* (INT38_GP) describing a *‘degree of fatalism and denial about cancer and perhaps more so with smokers’* (INT57_PH).

##### Belief in screening

Although some participants were cautious about screening, many described it as *‘a precaution’* (F5, FG65_FS_ED+) or that *‘prevention is better than cure’* (M6, FG63_CS_ED+), suggesting it *‘makes sense’* (F1, FG68_CS_ED−). Many participants recognised the benefits of early detection of cancers, and recognised screening as an ‘opportunity’, stating it was *‘silly’* to not *‘take advantage’* (F2, FG64_FS_ED+). Participants also described screening as *‘worthwhile’* due to its ability to *‘save lives’* (F1, FG68_CS_ED−).

This belief in screening, which was more frequently expressed by those with less education, appeared to be associated with a trust in medicine. Participants described attending screening because it was *‘recommended’* (M4, FG65_FS_ED+), or because *‘my doctor told me to do it’* (M2, FG67_FS_ED−). This phenomenon was reiterated by the HCPs.

Some participants seemed reluctant to acknowledge the harms of screening. They rationalised the harms by saying they were *‘so rare’* (F1, FG68_CS_ED-), or that a degree of inaccuracy when undergoing tests is unavoidable and could be overlooked *’nothing is 100%’* (F1, FG64_FS_ED+); and *‘there’s also human error in all this’* (F7, FG64_FS_ED+). HCPs described a need to dissuade people from tests at times, where the harm outweighed the benefits.

##### Right to an informed decision

Many focus group participants expressed the *‘human right’* (F1, FG63_CS_ED+) to be informed and to make an *‘individual choice’* (M4, FG65_FS_ED+) about participating. Participants, particularly current smokers and those with higher education, expressed a desire for information in order *‘to make an informed decision’* (M6, FG63_CS_ED+), and be better prepared for screening outcomes. Others were in favour of the decision being made on a population level, to avoid varying practices of different HCPs.

HCPs also acknowledged the ‘right’ to decide, and that people *‘want to know the facts and figures*’ (INT49_CNS) but that individual preferences varied. Some reported that balancing harms and benefits could be challenging particularly for *‘the group in the middle’* (INT38_GP) with whom it was most necessary for clinicians to spend time *‘not make the decision for people but trying to explain how to make that decision’* (INT38_GP).

##### Too much information

In contrast to the previous subtheme, a number of participants, most commonly current smokers, also expressed that at times *‘too much information’* (F8, FG64_FS_ED+) can be *‘too scary’* (F4, FG63_CS_ED+), or paralysing: *‘you can’t make any decision’* (M2, FG65_FS_ED+). Some participants referenced medical leaflets or information on the internet as a means to *‘frighten yourself to death’* (F1, FG68_CS_ED−) and some advocated placing *‘more emphasis on the positives’* (M6, FG63_CS_ED+) to mitigate this. Many participants, particularly those in the more educated groups expressed scepticism that statistics *‘can be played with’* (M6, FG64_FS_ED+) and are often manipulated, citing the phrase *‘lies, damn lies and statistics’* (M2, FG65_FS_ED+). Participant M2, FG65_FS_ED+ described his cynicism towards statistics presented by politicians, re-enforcing the idea that inclusion of too many statistics may be off-putting: *‘And if, if you look at some of, all the stuff coming out of, say, politicians’ mouths… when an election approaches, … each of them will use statistics, yeah, to prove quite different and disparate things’*. HCPs similarly highlighted that written information materials don’t always get read, and often resulted in *‘information overload*’ (INT58_CNS), and suggested it was necessary to moderate the information given.

#### Theme 2: reactions to the harms of LCS (table 3)

##### Anxiety associated with indeterminate nodules

There were mixed views on indeterminate nodules. Many participants, particularly current smokers, stated that being called back for repeated CT scanning could be *‘a worry’* (M8, FG63_CS_ED+) or *‘a concern’* (M6, FG63_CS_ED+). Some felt that *‘walking into hospital’* was *‘bad enough’* (F3, FG68_CS_ED−), or that the anxiety caused *‘in itself is bad for your health’* (M2, FG70_CS_ED+). Others suggested, *‘how you tell people’* (M2, FG65_FS_ED+) was important, and that being told the risk of cancer following an indeterminate result was low, could *‘make me feel a bit more confident and less worried*’ (M4, FG68_CS_ED−).

Some HCPs described circumstances where patients were *‘more worried than they need to be’* (INT40_GP). HCPs also described a challenge in communicating to patients that the *‘rate of that nodule being malignant is actually pretty low’* (INT51_RP), but that doing so was part of the role of the medical professional. Other HCPs felt it wasn't a *‘big problem for patients’* (INT45_RP) and that CT surveillance was a reassuring process for many.

##### False positives and negatives

A number of participants acknowledged the potentially *‘serious sequelae’* that may result from *‘interventions which might harm’* (M3, FG_70_CS_ED+) and some expressed concern that some people *‘wouldn’t be able to cope’* (M2, FG65_FS_ED+). Importantly, participants who had actually experienced false positives in other medical scenarios spoke of the *‘terrible fright’* (F1, FG68_CS_ED−) caused.

On the other hand, others suggested that they would find additional tests reassuring, as though *‘somebody’s looking after me’* (F4, FG63_CS_ED+). A number of participants felt false negatives were a far bigger worry *‘than the other way round’* (F4, FG67_FS_ED−), due to the fact that further tests for false positives could resolve the problem, while nothing could be done for missed cancers.

##### Overdiagnosis

Most focus group participants needed detailed explanation of the term ‘overdiagnosis’, though one participant who had a history of prostate cancer described the ‘tiger’ and the ‘sleepy’ cancers, and said *‘But if you get the tiger … you’re in trouble’* (M4, FG68_CS_ED−). Fear of cancer, perhaps accounted for why many felt *‘probably, it wouldn’t stop me being screened’* (M6, FG65_FS_ED+). Some participants were concerned about being *‘happy, smiley… and suddenly … get told, you’ve got cancer’* (M6, FG64_FS_ED+). Despite this, many felt they would rather know about the cancer and have the option not to treat it. When suggested that it may not always be possible to determine prognosis, participants felt *‘you can’t take that risk’* (F3, FG68_CS_ED−) of not treating. HCPs also acknowledged that patients often *‘don’t necessarily want to just say “oh leave it to be”’* (INT51_RP). One RP felt overdiagnosis was a *‘fallacy’* (INT72_RP) and supported the idea that expectant management of some ‘ground glass’ pulmonary nodules would reduce overdiagnosis.

##### Radiation exposure

The issue of radiation exposure generated some debate as it was acknowledged that *‘there’s no conclusion to be drawn from it because no one knows’* (M4, FG68_CS_ED−) because the exact harms from cumulative, medical doses were speculative. Some acknowledged that they knew very little about *‘x or radio, whatever it’s called’* (F2, FG64_FS_ED+), though overall most people felt it didn’t *‘worry me at all’* (F1, FG65_FS_ED+) or that *‘it’s a necessary thing unfortunately … And it’s not that bad’* (M8, FG63_CS_ED+), or that *‘the equipment nowadays is… much safer’* (M2, FG65_FS_ED+). Some did express some *‘concerns’* (F5, FG65_FS_ED+) due to having to go for repeat scans, while others felt it would not stop you having the test, *‘but it would be nice to know’* (M4, FG68_CS_ED−). HCPs placed different levels of importance on this harm. Some were concerned radiation was often ignored or caused much anxiety due to poor understanding, while others felt it was *‘doctors that are more concerned about that than the patients’* (INT45_RP).

## Discussion

In this study we used qualitative methods to investigate the opinions of LCS-eligible individuals and HCPs involved in the care of patients with lung cancer, focusing on the key features of lung screening, and in particular, the benefits and harms. These findings can enhance the development of information materials that may be acceptable to a broad range of individuals and be targeted to address the concerns highlighted. We found people at risk of lung cancer generally perceived it as an incurable and frightening condition, and smokers were particularly fatalistic. Despite this we found a persistent ‘belief in screening’, and appetite for information, with many participants expressing a ‘right’ to be fully informed, but others cautioning against too much information. False positives and false negatives were the harms that generated the most concern, but most participants were not deterred from screening.

Our finding that participants were generally unaware of curative treatment and thought it to be an incurable condition, is supported by previous studies that have shown fear, worry and fatalism about lung cancer^21–23^ as well as poor perceived benefit from LCS particularly among current smokers.^21 24^ A few participants were aware of positive outcomes in lung cancer, which may increase as more patients undergo curative treatment, but this finding demonstrates the need to provide information about curative treatment following LCS.

Our finding that participants and HCPs held a belief in screening as something that saves lives and expressed trust in medicine are supported in the literature.^25 26^ Thornton *et al*,^26^ reported that medical imaging is perceived as highly beneficial, though some contrasting studies have reported a variety of levels of trust of medical systems.^27 28^ In the face of this overriding trust in screening, we found a degree of disregard of the harms as either uncommon or insignificant, thus suggesting that providing information about the harms is unlikely to deter most individuals from LCS. This is supported by findings in two studies. One which demonstrated that individuals placed greater importance on LCS benefits than harms, particularly with respect to decision-making;^29^ and another UK based survey study reported that smokers welcomed the idea of LCS in principle.^30^

Participants had varying information preferences, with many feeling they had a ‘right’ to know and to make an informed decision, a finding that is supported by previous studies.^6 16 31^ Generally, it was accepted that policy makers should decide who screening should be offered to, and that the decision to participate should ideally be made by individuals with support from a medical professional if required. Other studies have similarly reported that autonomous decision-making with expert guidance is preferable.^32 33^ On the other hand, some participants were cynical or overwhelmed by ‘too much information’ and this variability in preferences makes designing decision literature and aids challenging. People have been shown to prefer personalised decision aids,^6^ and the limitations in literacy found in the at-risk population will make this even more important. For the core information materials we therefore propose that they should not be overly burdensome, with signposting to more detailed information.

Our finding that participants wanted to know about the potential harms of screening, even though these would be unlikely to deter them from participating, emphasises the importance of including these in information materials, consistent with other screening contexts.^5^ False negatives appeared to be of greater concern than false positives (in contrast to one other study^34^) and some people found additional investigations reassuring. Those who had encountered false positives and negatives were generally more concerned, suggesting that the hypothetical scenarios presented may understate people’s true reactions. These findings are very useful to inform the emphasis placed on the content of information; with the caveat that individuals do not feel reliant on further testing for reassurance when this is not advised clinically.

Awareness of overdiagnosis was low and the concept challenging to explain. However, most did not find this worrisome, either because they valued cancer treatment in spite of this issue or because they could opt for expectant management. Similar findings have been reported with respect to overdiagnosis in mammography screening.^16^ Radiation risk was poorly understood, although not a major deterrent. This is supported by a study investigating patients’ views on tests with ionising radiation, where many wanted to be made aware of the potential harms, however uncertain, while others found the uncertainty disconcerting and unhelpful.^26^

Anxiety as a result of indeterminate results, such as pulmonary nodules, was a concern for some participants which is supported by previous studies suggesting uncertainty associated with indeterminate nodules can weigh heavily on patients.^35^ However, many participants were reassured by the prospect of interval scanning and felt that psychological distress could be reduced by education around the low subsequent risk of developing cancer in the context of indeterminate pulmonary nodules. Studies have found individual differences in tolerance of uncertainty, that can affect how people weigh up benefits and risks, and that communication which effectively prepares patients for this likelihood, may mitigate poor tolerance of uncertainty^36^ and distress associated with pulmonary nodules.^37 38^

### Strengths and limitations

Selection bias is possible despite our attempts to mitigate this by purposively sampling varying educational and ethnic backgrounds. Despite this, it is likely that certain viewpoints may have been missed or under-represented or over-represented. HCPs were also included in the study design to provide insights that may have been missed by selection bias, and their data have significantly contributed to the structure and relative importance of the themes. Focus group participants discussed screening in the hypothetical sense and the fact that screening intentions are recognised to potentially differ from actual screening behaviours^39^ should be considered in the overall interpretation. Finally, the interviewer, who has a background in LCS and is an HCP, may have unintentionally biased the elicited data despite attempts to circumvent this (such as not disclosing her background, knowledge or any personal bias to the participants).

The strengths of the study include the wide range of demographic and educational backgrounds of participants. They were invited by a primary care database search in a similar way to what might occur in the setting of national LCS implementation, and almost all the participants would qualify for LCS if offered according to the USPSTF recommendations. Furthermore, the lack of availability and public knowledge in the UK for LCS has enabled recruitment of a group of individuals with no prior knowledge of LCS and pre-existing external biases. The findings from this study have helped inform the content and format of an information film designed to promote informed decision-making in LCS-eligible individuals, and has been subsequently tested as a nested randomised study within the Lung Screen Uptake Trial.^9^ The film is available to view via the Roy Castle Lung Cancer Foundation website (https://www.roycastle.org/lungcancerscreeningguide).

## Conclusions

Addressing the information needs of the whole screening-eligible population in a way that meets diverse information preferences is challenging. Policy makers need to ensure LCS information materials are effective in helping individuals comprehend complex risks and benefits in a way that addresses the concerns and preferences found here. In particular, our findings suggest that information materials are best presented simply, and should direct participants to more detailed information if preferred, and not replace the support of an HCP. Additional work should be carried out to further explore how we might effectively support more conflicted individuals reach an informed decision that is in line with their values and beliefs, and to test the impact of targeted information materials on informed decision-making.

## References

[1] Aberle DR, Adams AM, Berg CD, et al Reduced lung-cancer mortality with low-dose computed tomographic screening. N Engl J Med 2011;365:395–405. 10.1056/NEJMoa1102873 21714641 PMC4356534

[2] Koning HJD, Carlijn M, Aalst VD, et al Effects of volume CT lung cancer screening. mortality results of the Nelson randomised controlled population-based screening trial. world Congr lung cancer, 2018 Available: https://library.iaslc.org/virtual-library-search?product_id=10&author=&category=&date=&session_type=&session=&presentation=&keyword=NELSON

[3] Pastorino U, Silva M, Sestini S, et al Prolonged lung cancer screening reduced 10-year mortality in the mild trial: new confirmation of lung cancer screening efficacy. Ann Oncol 2019;30:1162–9. 10.1093/annonc/mdz117 30937431 PMC6637372

[4] Bach PB, Mirkin JN, Oliver TK, et al Benefits and harms of CT screening for lung cancer. JAMA 2012;307 10.1001/jama.2012.5521 PMC3709596 22610500

[5] Hersch JK, Nickel BL, Ghanouni A, et al Improving communication about cancer screening: moving towards informed decision making. Public Health Res Pract 2017;27:2731728 10.17061/phrp2731728 28765861

[6] Crothers K, Kross EK, Reisch LM, et al Patients' attitudes regarding lung cancer screening and decision AIDS. A survey and focus group study. Ann Am Thorac Soc 2016;13:1992–2001. 10.1513/AnnalsATS.201604-289OC 27652509 PMC5466178

[7] Centers for Medicare & Medicaid Services Decision MEMO for screening for lung cancer with low dose computed tomography (LDCT) (CAG-00439N), 2015 at Available: http://www.cms.gov/medicare-coverage-database/details/nca-decision-memo.aspx?NCAId=274

[8] The UK NSC recommendation on lung cancer screening. Available: http://www.screening.nhs.uk/lungcancer

[9] Quaife SL, Ruparel M, Beeken RJ, et al The Lung Screen Uptake Trial (LSUT): protocol for a randomised controlled demonstration lung cancer screening pilot testing a targeted invitation strategy for high risk and 'hard-to-reach' patients. BMC Cancer 2016;16:281 10.1186/s12885-016-2316-z 27098676 PMC4839109

[10] Crosbie PA, Balata H, Evison M, et al Implementing lung cancer screening: baseline results from a community-based 'Lung Health Check' pilot in deprived areas of Manchester. Thorax 2019;74:405–9. 10.1136/thoraxjnl-2017-211377 29440588

[11] Targeted screening for lung cancer with low radiation dose computed tomography: standard protocol prepared for the targeted lung health checks programme, 2019 Available: www.england.nhs.uk/cancer

[12] Fagerlin A, Zikmund-Fisher BJ, Ubel PA Helping patients decide: ten steps to better risk communication. J Natl Cancer Inst 2011;103:1436–43. 10.1093/jnci/djr318 21931068 PMC3218625

[13] Jepson RG, Hewison J, Thompson A, et al Patient perspectives on information and choice in cancer screening: a qualitative study in the UK. Soc Sci Med 2007;65:890–9. 10.1016/j.socscimed.2007.04.009 17507131

[14] Ghanouni A, Meisel SF, Renzi C, et al Survey of public definitions of the term ‘overdiagnosis’ in the UK. BMJ Open 2016;6:e010723 10.1136/bmjopen-2015-010723 PMC4838699 27053274

[15] Hersch J, Jansen J, Barratt A, et al Women's views on overdiagnosis in breast cancer screening: a qualitative study. BMJ 2013;346:f158 10.1136/bmj.f158 23344309 PMC3552499

[16] Waller J, Douglas E, Whitaker KL, et al Women's responses to information about overdiagnosis in the UK breast cancer screening programme: a qualitative study: Table 1. BMJ Open 2013;3:e002703 10.1136/bmjopen-2013-002703 PMC3641428 23610383

[17] Department for Business Innovation and Skills The 2011 skills for life survey: a survey of literacy, Numeracy and ICT levels in England, 2012 Available: https://www.gov.uk/government/publications/2011-skills-for-life-survey

[18] Public Health England and Cancer Research UK Cancer by deprivation in England incidence, 1996-2010 mortality, 1997-2011, 2014 Available: http://www.ncin.org.uk/about_ncin/cancer_by_deprivation_in_england

[19] Gale NK, Heath G, Cameron E, et al Using the framework method for the analysis of qualitative data in multi-disciplinary health research. BMC Med Res Methodol 2013;13:117 10.1186/1471-2288-13-117 24047204 PMC3848812

[20] Ritchie J, Lewis J Qualitative research practice: a guide for social science students and researchers. Sage Publications, 2003 https://books.google.co.uk/books?hl=en&lr=&id=z5y0LCT8YNUC&oi=fnd&pg=PA219&ots=q4aPFjI0G1&sig=Rp2Vil8EESlf8gpB0BYkvpIay3g&redir_esc=y#v=onepage&q&f=false

[21] Quaife SL, Marlow LAV, McEwen A, et al Attitudes towards lung cancer screening in socioeconomically deprived and heavy smoking communities: informing screening communication. Health Expect 2017;20:563–73. 10.1111/hex.12481 27397651 PMC5513004

[22] Park ER, Streck JM, Gareen IF, et al A qualitative study of lung cancer risk perceptions and smoking beliefs among National lung screening trial participants. Nicotine Tob Res 2014;16:166–73. 10.1093/ntr/ntt133 23999653 PMC3934998

[23] Farley A, Aveyard P, Kerr A, et al Surgical lung cancer patients' views about smoking and support to quit after diagnosis: a qualitative study. J Cancer Surviv 2016;10:312–9. 10.1007/s11764-015-0477-4 26298019

[24] Silvestri GA, Nietert PJ, Zoller J, et al Attitudes towards screening for lung cancer among smokers and their non-smoking counterparts. Thorax 2007;62:126–30. 10.1136/thx.2005.056036 17101739 PMC2111262

[25] Waller J, Osborne K, Wardle J Enthusiasm for cancer screening in Great Britain: a general population survey. Br J Cancer 2015;112:562–6. 10.1038/bjc.2014.643 25535731 PMC4453657

[26] Thornton RH, Dauer LT, Shuk E, et al Patient perspectives and preferences for communication of medical imaging risks in a cancer care setting. Radiology 2015;275:545–52. 10.1148/radiol.15132905 25803490 PMC4976442

[27] Gressard L, DeGroff AS, Richards TB, et al A qualitative analysis of smokers’ perceptions about lung cancer screening. BMC Public Health 2017;17:589 10.1186/s12889-017-4496-0 28637439 PMC5479014

[28] Young B, Bedford L, Kendrick D, et al Factors influencing the decision to attend screening for cancer in the UK: a meta-ethnography of qualitative research. J Public Health 2017:1–25. 10.1093/pubmed/fdx026 28486650

[29] Lillie SE, Fu SS, Fabbrini AE, et al What factors do patients consider most important in making lung cancer screening decisions? findings from a demonstration project conducted in the Veterans health administration. Lung Cancer 2017;104:38–44. 10.1016/j.lungcan.2016.11.021 28212998

[30] Quaife SL, Vrinten C, Ruparel M, et al Smokers' interest in a lung cancer screening programme: a national survey in England. BMC Cancer 2018;18:497 10.1186/s12885-018-4430-6 29716550 PMC5930691

[31] Kanodra NM, Pope C, Halbert CH, et al Primary care provider and patient perspectives on lung cancer screening. A qualitative study. Ann Am Thorac Soc 2016;13:1977–82. 10.1513/AnnalsATS.201604-286OC 27676369

[32] Waller J, Macedo A, von Wagner C, et al Communication about colorectal cancer screening in Britain: public preferences for an expert recommendation. Br J Cancer 2012;107:1938–43. 10.1038/bjc.2012.512 23175148 PMC3516693

[33] Hopmans W, Damman OC, Senan S, et al A patient perspective on shared decision making in stage I non-small cell lung cancer: a mixed methods study. BMC Cancer 2015;15:959 10.1186/s12885-015-1974-6 26673216 PMC4682255

[34] Carter-Harris L, Brandzel S, Wernli KJ, et al A qualitative study exploring why individuals opt out of lung cancer screening. Fam Pract 2017;34:239–44. 28122849 10.1093/fampra/cmw146 PMC6279209

[35] Wiener RS, Gould MK, Woloshin S, et al 'The thing is not knowing': patients' perspectives on surveillance of an indeterminate pulmonary nodule. Health Expect 2015;18:355–65. 10.1111/hex.12036 23252477 PMC3880393

[36] Schapira MM, Aggarwal C, Akers S, et al How patients view lung cancer screening. The role of uncertainty in medical decision making. Ann Am Thorac Soc 2016;13:1969–76. 10.1513/AnnalsATS.201604-290OC 27676595 PMC5122480

[37] Slatore CG, Wiener RS Pulmonary nodules: a small problem for many, severe distress for some, and how to communicate about it. Chest 2018;153:1004–15. 10.1016/j.chest.2017.10.013 29066390 PMC5989642

[38] Slatore CG, Wiener RS, Golden SE, et al Longitudinal assessment of distress among veterans with incidental pulmonary nodules. Ann Am Thorac Soc 2016;13:1983–91. 10.1513/AnnalsATS.201607-555OC 27599153

[39] Sheeran P Intention—Behavior relations: a conceptual and empirical review. Eur Rev Soc Psychol 2002;12:1–36. 10.1080/14792772143000003

