# Correction: *Defining the information needs of lung cancer screening participants: a qualitative study*

**DOI:** 10.1136/bmjresp-2019-000448corr1

**Published:** 2019-12-30

Ruparel M, Quaife S, Baldwin D, *et al*. Defining the information needs of lung cancer screening participants: a qualitative study. *BMJ Open Respir Res* 2019;6:e000448. doi: 10.1136/bmjresp-2019-000448.

The license type of the paper has changed from CC BY-NC to CC BY.

