# Supplementary material for: Defining the information needs of lung cancer screening participants: a qualitative study
Source: BMJ Open Respir Res. 2019 Nov 24;6(1):e000448. doi: 10.1136/bmjresp-2019-000448 (PMC6890387; doi:10.1136/bmjresp-2019-000448)
Supplement: Supplementary data [file bmjresp-2019-000448supp001.pdf]

Supplementary Appendix

1. Focus group discussion guide
2. Health care professional interview schedule
3. Table S1 – further quotes illustrating the ‘Appetite for balanced information’ theme
4. Table S2 – further quotes illustrating the ‘Reactions to the harms of LCS’ theme

Ruparel M, *et al. BMJ Open Resp Res* 2019; 6:e000448. doi: 10.1136/bmjresp-2019-000448

DISCUSSION GUIDE: FOCUS GROUPS

Introduction/Ethics/Consent

- Greet participants as they arrive and take consent for participation in the study.
- Allow participants to take refreshments and then start the focus groups once all have arrived. Then, open with introduction below.
- The reason I'd like to talk with you today is to find out about your knowledge and experience of cancer screening and lung cancer.
- I am carrying out a number of these group sessions to get an overview from different individuals.
- I do understand that some people may not feel comfortable talking about cancer or may have had experiences that can be difficult to talk about. If at any time you are not feeling comfortable, please let me know.
- Does any one have any concerns about any of that so far? [*Option of going away and thinking about it, or declining*]
- So that I do not need to take notes, I will record our conversation using this device. This will later be typed up and I will delete the recording. Everything you say will remain strictly confidential and completely anonymous. Other researchers may read our conversation, but nobody else. I hope to write a report of my findings to publish in a scientific journal. This would summarise opinions from everyone I have spoken to and may include some of your quotes. However, your name will never be included, and you will not be identifiable in any way.
- I have a few questions noted down here of things I'd like to ask you but I'd like you to do most of the talking.
- I want to hear your views so please feel free to be as frank and open as you wish. There are no right or wrong answers. We don't have to talk about anything that you feel uncomfortable about and you are free to withdraw at any time, without giving a reason.
- I expect the session to last between 60 and 90 minutes, but if you need to leave, please let me know

MAIN DISCUSSION POINTS

NHS CANCER SCREENING PROGRAMMES

1. Discuss what experiences they have had with other NHS cancer screening programmes.
   a. Purpose of screening.
   b. Reasons for going for screening (or not).
   c. Are they aware of any possible drawbacks or harms from screening?
   d. Do they feel taking part in screening is a positive thing to do for their health?

LUNG CANCER

2. What have they heard about lung cancer and its treatments?
   a. What it is and how it is caused.
   b. How frequently it occurs in the general population.
   c. Have they had any experience of lung cancer in anyone you know?
   d. How curable it is, and what affects curability.
   e. What sorts of treatments do you think are available for lung cancer?
   f. How long might someone with lung cancer expect to live?

LUNG CANCER SCREENING

Interviewer: "Research in the US has shown that if we carry out a CT scan (a detailed sort of X-ray) once a year on people who have a higher risk of lung cancer due to the amount they have smoked in the past, we

Ruparel M, *et al. BMJ Open Resp Res* 2019; 6:e000448. doi: 10.1136/bmjresp-2019-000448

may save 20% of lives by detecting the cancer early and giving a higher chance of cure. There are more trials underway, and depending on the results of those, we may start doing lung cancer screening in the UK in a few years. As with the other screening programmes we have discussed, there are pros and cons to screening for lung cancer. Here are some leaflets on lung cancer screening. I will give you some time to read through them and then, if it's ok, I'll ask you for your thoughts on them."

3. Explore views on the contents of the leaflets.
    a. Initial thoughts / reactions to the leaflet
    b. Discuss radiation, over diagnosis, false positives.
    c. Discuss their feelings around having tests and treatments that may later prove to have been unnecessary vs potential to save life by early diagnosis. Do these harms influence their decision of whether to be screened?
    d. Discuss the benefits of reassurance from a normal result.
    e. Discuss understanding around numeric data [2% detection rate, 25% indeterminate results]
    f. Who should decide if screening is the right thing to do? How would their decision be influenced by the health professional's opinion?

RESPONSE TO DECISION AIDS

We are planning to make a short video that could explain the concepts we have discussed today that would help people considering having lung cancer screening make a decision of whether or not to be screened. I would therefore be grateful for your opinions and feelings about some written materials or clips from films used for the same purpose in lung and other screening programmes and also some of the ideas we have thought about for our film.

4. Gauge thoughts on:
    a. Leaflet used in UCL lung cancer screening pilot: "lung health check – information on what's involved" and similar bowel and breast screening leaflets.
    b. Show general video clips, pictures or read vignettes, and get opinions and thoughts as above
        i. Especially what they feel about the methods to explain concepts such as metaphors or numeric person indicators.
        ii. Do they help to make a decision one way or the other?
        iii. Do they convey all the facts we discussed today? If not, what is not clear that they think is important?
        iv. What ideas do they have that they think would make them feel more empowered to do positive things to impact their health- eg scenes, metaphors, messages, ideas?
    c. Discuss (and view if available) concepts from creative team.
    d. Discuss the idea of health professional speaking or a person speaking about personal experiences and who should be conveying what facts?
    e. What should we say about smoking if anything?

CLOSE AND DEBRIEF

- Is there anything else you would like to mention that you feel we haven't covered?
- I really appreciate your time today and thank you very much for sharing your views with me, these are really interesting and helpful. We are hoping to have some more ideas or clips from our produced film that we would like to show you and get feedback on in 4-6 weeks time. Could you please let me know if you are happy to be invited back for this?
- Thank participants for their time and input and reassure about confidentiality.
- Answer any questions and provide with debrief information sheet and researcher contact details.
- Offer copy of transcript and report.

INTERVIEW SCHEDULE: CLINICIAN INTERVIEWS

The following questions are intended as a rough guide. The questions chosen, and the order in which they are asked, will depend on responses to preceding questions.

Introduction/Ethics/Consent

“The reason I’d like to talk with you today is because we are trying to produce an information film to help people considering undergoing lung cancer screening make an informed decision. This will be in the form of a short information film (3-5 minutes long). As you have experience with diagnosing/ looking after patients with lung cancer, we would be very grateful for you thoughts and opinions. I will ask questions but I’d like you to do most of the talking. It should take about 20-30 minutes depending on how much time you have.”
“I will record our conversation and it will be typed up later. Everything you say will remain strictly confidential and anonymous. I hope to include my findings which may include some anonymised quotes in my thesis and in publications in scientific journals. Would that be ok?”

MAIN INTERVIEW QUESTIONS

---

1. What do patients/ individuals know about lung cancer? Can they give me some examples of experiences they have had with patients who have been newly diagnosed with lung cancer or indeterminate pulmonary nodules?
    - Can you tell me what you usually tell patients about lung cancer and/or indeterminate nodules?
    - Can you tell me what questions people usually ask?
    - Do they find any particular concepts difficult to understand?
    - Do you find anything particularly challenging to communicate to them? How do you get around it?
    - Do you think patients have an accurate idea of curability and risk?
    - When you talk about stage, what do they understand by it?
    - Do they understand how stage implicates treatments and prognosis?
2. Imagine you are carrying out screening in your institution.
    - Can you think of patients that you know that may have made an informed decision not to be screened?
    - If so, what concerns do you think they might have about the screening process? Do you think it may be based on true fact or mis-information/ misconceptions?
    - If they have patients who they think might not have chosen to be screened? Can they tell me more about them?
3. Can they give examples of patients where they have had to consider or discuss issues such as overdiagnosis and surgical risk (operability vs. resectability)? What worked well? What worked badly? Give example of pulmonary nodules.
4. What concepts/ facts about screening do you think are most important for patients to understand?
5. What techniques do they use to explain things? Examples of metaphors.
6. Check response to:
    - Patients giving experience of lung cancer / treatment
    - Clinician/ expert explaining lung cancer screening
    - Smoking cessation
    - Concepts from film company
    - Examples of other decision aids- what works well and what doesn’t

CLOSE AND DEBRIEF

"Is there anything else you would like to mention that you feel we haven't covered?"

"I really appreciate your time today and thank you very much for sharing your views with me, these are really interesting and helpful. Based on what we have learned from interviewing yourself and other experts as well as from members of the public that would be eligible for screening, we will be developing the ideas and a script for the final information film. Would you mind if we send you a summary of these ideas in a few weeks time and get you to fill in a feedback form on your thoughts on what in your opinion works well and what doesn't?"

Answer any questions and provide with debrief information sheet and researcher contact details. Offer copy of transcript and report. Thank participant and end interview.

Ruparel M, *et al. BMJ Open Resp Res* 2019; 6:e000448. doi: 10.1136/bmjresp-2019-000448

Table S1. Quotes illustrating the 'Appetite for balanced information' theme

Fatalism and perception of lung cancer as incurable	
"I didn't think there was any [treatment]"	M6, FG63_CS_ED+
"I don't know much. I had a friend that got it and he died but they kept him going for quite some time somehow … they told him he was going to die within six months and he got probably about three years"	M4, FG68_CS_ED-
"Well if you hear that someone's got lung cancer, you know, you, you do feel that it's not a good prognosis. Put it that way"	F8, FG64_FS_ED+
"it's the big C word to the general populous, isn't it? … and the minute you mention that word, everything is invasive and almost terminal. … it's the worst possible scenario for most people to hear they've got cancer"	F5, FG70_CS_ED+
"if you haven't met many people who've had surgery for lung cancer… whereas … you find people saying well I had breast cancer and I'm cured"	INT61_GP
"Yeah I think most people have known somebody who's died of it rather than survived from it"	INT54_CNS
"As treatments have got better again there will be a certain amount of day to day experience of knowing people who have had it for longer … but… there probably is the perception that it's quite a bad one to get"	INT57_PH
"I think [for patients] cancer equals, you know death, chemotherapy, suffering all those, all those bad things"	INT50_GP
"I think probably a lot of their knowledge comes from people they already know who have had lung cancer and because of its frequent terminal course, they're very frightened of the diagnosis of lung cancer"	INT55_GP
Belief in screening	
**Early detection/ life saving**	
"I did believe in having the screening because if cancer's caught early enough … you can battle"	F5, FG67_FS_ED-
"But that [bowel screening], that's all that men get isn't it, really?"	M6, FG63_CS_ED+
**Trust in medicine**	
"I trust the consultant … why would he put me through this if he thinks it's a waste of time and a waste of National Health money"	F4, FG63_CS_ED+
"most of the patients … they're happy to do whatever the doctor thinks is right and actually it doesn't really matter what you say to them, you could tell them there's a 99% risk of death but if you recommended it to them they'd still want to have it done"	INT45_RP
"I think that patients had … cancer screening programme fully explained to them I, I suspect they might still have an exaggerated view of those benefits because people want to believe that the screening is good for you"	INT52_PH
**Harm rationalisation**	
"Well yeah, I mean I know that some people say that they've got mistaken results … and it's caused them great distress. But, you know, I think that must be the minority of what happens and as such I can't see it to be a reason for not doing it"	F2, FG64_FS_ED+
"But all the tests you can make are not infallible … mistakes can be made"	M2, FG65_FS_ED+
"If it was very common it might change my mind but I think you only hear about the ones that are false negatives or false positives rather than the thousands and millions of tests where they get it right ... So it wouldn't put me off"	F3, FG68_CS_ED-
"I think if there's a large amount, that if it was common, they would, they would find what was going wrong … And put it right"	M4, FG68_CS_ED-

The right to an informed decision	
"I would look into it and find out a bit more about it … to put my own mind at rest … And to know what's going on"	M4, FG68_CS_ED-
"I would like to know exactly what's going on, all the test and the results and everything and then I make my own conclusion"	F2, FG63_CS_ED+
"I think that if you decide that a set of people should be screened for lung cancer … then you should say to everybody in, in that sort of risk of category … But I don't think that, say, an individual doctor, or whatever, should … Make the decision for you"	M2, FG65_FS_ED+
"I tend to probably express it… there are further consequences and, but actually if you really are at risk, or you want to know the result, then this is the next step, and you need to be aware that this is what could be coming next"	INT53_GP
"[we] would wish patients to be given a totally impartial degree of information so … they did understand that … you get interval cancers … sometimes things do go wrong"	INT52_PH
Too much information	
"When I had my babies I didn't even think about a miscarriage or percentages or this, that and the other. But now they scare them, they scare the mothers to death and other people, because they give out too much information"	F4, FG63_CS_ED+
"the egg crisis, the beef crisis, didn't stop me. … If you look at them, it's all, this is bad for you, that's bad for you, I mean, now I mean, they're, they're, they're on about sugar… I'm very cynical in a lot of things because I don't trust what a lot of people say"	M2, FG69_CS_ED_
"I think trying to read a black and white document is very difficult… But it's just such a lot of information that I think people do struggle"	INT53_GP
"so it's not trying to undermine the risks but … you're not scaring them as well"	INT42_GP

7

Table S2. Quotes illustrating the 'Reactions to the harms of LCS' theme

Anxiety associated with indeterminate results	
"It wasn't saying that there was a problem. It was just saying inconclusive. But even so, you think, oh, yeah"	F5, FG70_CS_ED+
"whatever you're told you still worry. I'm, you know, a terrible worrier. But I keep saying to myself, don't worry about things until you've got something to worry about. But, you know, you can't help it."	M6, FG64_FS_ED+
"if it can be resolved quickly then I suppose it's not pleasant but it's less of a worry, but if … that could be a prolonged period of time that people could be quite distressed"	INT38_GP
False positives and negatives	
"I'm thinking I'd rather be one of those thirty false positives out of the thousand and they do something"	F5, FG64_FS_ED+
"I was told … it's just a polyp … And it wasn't a polyp … it was a tumour … he … could have said … well I think it's a tumour. Not, not, oh don't worry about it, it's just a polyp"	M6, FG64_FS_ED+
"I certainly think that anybody who is flagged as suspicious and requires a further intervention, I think that is a much greater harm than the others, and partly psychological but they also undergo that physical harm as well"	INT46_PH
"False negatives I would think would be a concern. False positives are a concern but as long as it's presented to patients in a way that … it isn't normal but it might not be anything and … it's dealt with in a timely fashion then I guess people probably would cope with it reasonably well"	INT38_GP
Overdiagnosis	
"so I could go through life completely having a lovely time, happy, smiley, enjoy my life and suddenly I get told, you've got cancer"	M6, FG64_FS_ED+
"somebody giving me a sound sort of prognosis… like saying, well you could actually live with this for X number of years … or they will say, well look we think this is serious and it could spread and so on"	F1, FG64_FS_ED+
"But at that stage would they give you the choice then whether to, to do something or not? … there's the other side of it … where things aren't done … And weren't acted upon quickly enough"	F3, FG68_CS_ED-
Radiation exposure	
"Well it doesn't worry me. You know, it's just one of those things that have got to be done"	M6, FG64_FS_ED+
"I think radiation is particularly difficult because people are, either just ignore it because they don't understand it or they become very, very anxious about it because they don't understand it"	INT46_PH
"You don't very often hear patients worrying about radiation doses"	INT38_GP
"I think across the board that [radiation] is the least worry thing, worrisome thing for them"	INT72_RP
"I wonder if they've overplayed the radiation risk sometimes"	INT75_RP
"I think most people's perception of radiation is oh well, I'll trust you doc, it's a low dose, it's worth the risk"	INT61_GP
"Some people are aware of radiation but others, now with scanning … so frequent nowadays, you go in and you have a CT scan for goodness knows what, it seems to be part and parcel of it … I'm not sure whether or not people a) can understand or b) how one gets that across to people in a way that they can understand and make that decision"	*INT61_GP*
